# Enhancing Oocyte Competence With Milrinone as a Phosphodiesterase 3A Inhibitor to Improve the Development of Porcine Cloned Embryos

**DOI:** 10.3389/fcell.2021.647616

**Published:** 2021-04-30

**Authors:** Pantu Kumar Roy, Ahmad Yar Qamar, Bereket Molla Tanga, Xun Fang, Ghangyong Kim, Seonggyu Bang, Jongki Cho

**Affiliations:** ^1^Laboratory of Theriogenology, College of Veterinary Medicine, Chungnam National University, Daejeon, South Korea; ^2^College of Veterinary and Animal Sciences, Jhang, Sub-campus of University of Veterinary and Animal Sciences, Lahore, Pakistan; ^3^Faculty of Veterinary Medicine, Hawassa University, Hawassa, Ethiopia

**Keywords:** oocyte, cloning, development, meiosis, milrinone

## Abstract

The objective of this study was to investigate the effect of milrinone supplementation as a phosphodiesterase 3A inhibitor during *in vitro* maturation (IVM) to coordinate the cytoplasmic and nuclear maturation of porcine oocytes and subsequent development of porcine cloned embryos. Brilliant cresyl blue (BCB)-stained (BCB +) oocytes, classified as well-developed, and BCB− oocytes were used in parthenogenesis (PA) and cloning, and their preimplantation development was compared. In PA embryos, BCB + oocytes had significantly higher rates of development than BCB− oocytes in terms of maturation (87.5 vs. 71.3%), cleavage (88.6 vs. 76.3%), and blastocyst development (34.3 vs. 25.3%) and also had higher cell numbers (46.9 vs. 38.9%), respectively (*p* < 0.05). In cloned embryos, the BCB + group also had a significantly higher blastocyst formation rate than the BCB− group (30.6 vs. 20.1%; *p* < 0.05). Supplementation with 75 μM milrinone during IVM of BCB− oocytes showed improvement in maturation and blastocyst development rates, which may be due to the coordinated maturation of the cytoplasm with the nucleus as an effect of milrinone. Moreover, the analysis of nuclear reprogramming via the examination of the expression levels of the reprogramming-related genes *POU5F1*, *DPPA2*, and *NDP52IL* in milrinone-supplemented BCB− oocytes showed higher expression levels than that in non-treated BCB− oocytes. These findings demonstrate that milrinone is useful in improving developmental competence in less competent oocytes during IVM and for proper nuclear reprogramming in the production of porcine cloned embryos by coordinating cytoplasmic and nucleus maturation.

## Introduction

*In vitro* production (IVP) of embryos has low efficiency, particularly for cloned embryos, which is mainly attributed to poor competence of oocytes during *in vitro* maturation (IVM) (Manjunatha et al., 2007). The importance of selecting competent oocytes that have high development rates and can result in the successful establishment of pregnancy has been reported in previous studies (Mehlmann et al., 2002; Gilchrist, 2011). Although many factors, such as limitations in culturing conditions and the stage of the donor cell cycle, have been identified as affecting the efficiency of embryo production, the ability to attain competent oocytes in IVM remains the main obstacle (Hunter, 2000). Conventionally, the developmental competence of an oocyte is generally affected by the age of the animal (Armstrong, 2001), follicular size (Trounson et al., 2001), oocyte size (Durinzi et al., 1995), and oocyte quality (Karami-Shabankareh and Mirshamsi, 2012). Furthermore, Russell (1998) found that there is a positive relationship between oocyte diameter and the ability of the oocyte to develop to the blastocyst stage. Consequently, it has been proposed that follicular size and oocyte diameter can be used as selection parameters (Roca et al., 1998). Nowadays, developmental competence of an IVM oocyte refers to the coordinated maturation of the cytoplasm and nucleus in a controlled manner, attaining the full development of the oocyte, similar to that in the *in vivo* state, which supports the success of subsequent fertilization and embryo development (Nogueira et al., 2006).

Before luteinizing hormone (LH) surge for ovulation, oocyte cytoplasm is growing and fully developed during arrest in prophase 1 due to the effect of cyclic nucleotides, which are mainly synthesized by the surrounding granulosa cells (Gupta et al., 2017). Nuclear maturation is resumed by LH surge after sufficient development of the ooplasm. In IVM, as oocytes are aspirated from the follicles, there is a spontaneous resumption of meiosis, leading to an uncoordinated nuclear and cytoplasmic maturation. This uncoordinated maturation, in turn, leads to less developmental competence, resulting in a poor rate of blastocyst development (Combelles et al., 2002). The role of cyclic nucleotides in arresting the oocyte from meiosis is primarily regulated by nucleotide phosphodiesterase (PDE) enzymes, which hydrolyze these cyclic nucleotides and lead to a decrease in cyclic adenosine monophosphate (cAMP) and cyclic guanosine monophosphate (cGMP) levels, resulting in the resumption of meiosis (Azevedo et al., 2014; Gupta et al., 2017). The development of oocytes is also affected by granulosa cells that produce cAMPs and transfer them to the oocyte through gap junctions, allowing high levels of cAMP to be maintained (Bilodeau-Goeseels, 2011). Accordingly, the application of PDE inhibitors in IVM has shown promising results in improving the competence of oocytes in terms of better coordination of cytoplasmic and nuclear maturation in different species of animals, including humans (Taiyeb et al., 2014; Gupta et al., 2017; Leal et al., 2018). To improve the coordination of cytoplasmic and nuclear maturation during IVM, PDE inhibitors such as caffeine, theophylline, and cilostazol have been applied, yielding progressive results for IVP in different species (Elahi et al., 2016; Gupta et al., 2017; Taiyeb et al., 2020).

Developmentally competent oocytes with sufficient ooplasmic maturation are more necessary in somatic cell nuclear transfer (SCNT), which exhibits aberrant gene expression compared to *in vitro* fertilization (IVF) embryos, owing to incomplete reprogramming of the donor nuclei (Zhang et al., 2009). This fact is evident from several reports, which demonstrate that genomic reprogramming in the cloning of mice was affected by the level of cytoplasmic development of the oocytes, a key factor in cloning (Bui et al., 2008). Nuclear reprogramming was thought to be determined only by the donor nucleus, but it has been demonstrated that the role of the recipient oocyte cytoplasm is the most critical determining factor for the success of SCNT (Kang et al., 2014). Thus, a fully developed recipient ooplasm, which is in coordinated meiosis, is the determining factor for proper reprogramming in SCNT.

In this study, we aimed to improve oocyte competence by coordinating maturation, thereby improving the efficiency of cloned pig production by SCNT. The efficiency of reprogramming can be assessed by evaluating gene transcription as an indication of proper embryonic development and correct reprogramming of donor nuclei (You et al., 2010; Fang et al., 2018). For this purpose, we evaluated the application of specific PDE3A inhibitors to improve coordinated oocyte maturation, ultimately improving the efficiency of porcine SCNT. Milrinone has improved of the *in vitro* maturation of oocytes in different species, which improved the success in IVF, bovine (Alam et al., 2018), in ovine (Wang et al., 2016) oocyte maturation. The role of proper cytoplasmic maturation is believed to be important in nuclear reprogramming, we propose the application of PDE3A for improving efficiency of cloning in porcine. We used milrinone, a PDE3A inhibitor, to improve the developmental competence of recipient oocytes via the induction of coordinated maturation of the nucleus and ooplasm in less competent oocytes, which were classified by Brilliant cresyl blue (BCB) staining. We evaluated the effect of milrinone on the developmental competence of porcine cloned embryos and calculated the reprogramming efficiency by assessing the expression of *POU5F1*, *NDP52I1*, and *DPPA2* genes (Boiani et al., 2002; Loh et al., 2006), as an indicator of the efficiency of nuclear reprogramming.

## Materials and Methods

### Chemicals and Reagents

All chemicals and reagents were purchased from Sigma-Aldrich (St. Louis, MO, United States) unless otherwise indicated.

### IVM and Selection of Competent Oocytes

**FIGURE 1:**
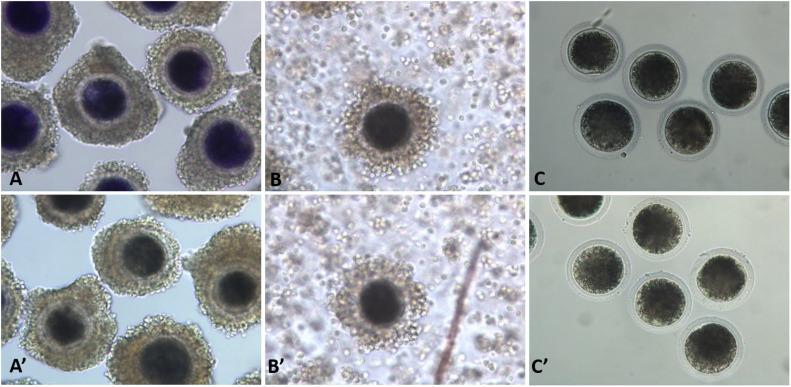
Porcine oocytes after brilliant cresyl blue (BCB) staining. BCB−stained (BCB+; **A–C**) and non-stained (BCB−; **A′–C′**) oocytes in the immature cumulus–oocyte complexes (COCs; **A,A′**), mature COCs (**B,B′**), and denuded mature oocytes (**C,C′**).

Pig ovaries were collected from a local slaughterhouse within 4 h of slaughter. The follicular fluid of follicles with diameters ranging from 3 to 8 mm was aspirated using a 10-mL syringe with an 18-ga needle. Fluids were collected in 15-mL conical tubes and oocytes were settled within 5 min and then washed with HEPES-buffered Tyrode’s (TLH) medium containing 0.05% (wt/vol) polyvinyl alcohol (PVA). Cumulus–oocyte complexes (COCs) were washed three times with TLH-PVA, and those with at least three layers of compact cumulus cells were selected. The COCs were then exposed to 13 μM BCB (B-5388) supplemented with modified Dulbecco’s phosphate-buffered saline (mDPBS) with the addition of 0.4% BSA for 90 min at 38.5°C in a humidified atmosphere of 5% CO_2_ and 95% air (Roy et al., 2017). The COCs were exposed to BCB, washed twice in mDPBS, and classified as BCB + (if cytoplasm stained blue) or BCB− (if cytoplasm colorless/without blue stain) oocytes (Figure 1). In addition, control oocytes were prepared by washing COCs three times and placing them immediately in the IVM medium without BCB staining. Mature oocytes were denuded after 44 h of incubation in IVM medium and their diameters were measured under a microscope (200 × magnification) using Leica Application Suite X (LAS X) (Wetzlar, Germany). Glutathione (GSH) and reactive oxygen species (ROS) levels were measured as described in our previous studies (Roy et al., 2020a,b).

### Production of Embryos

Porcine parthenogenetic and SCNT embryos were produced as described in our previous studies (Fang et al., 2018; Kim et al., 2019). To produce parthenogenetic embryos, COCs were cultured in IVM medium for 44 h and then placed in IVM medium supplemented with 0.1% (w/v) hyaluronidase. The cumulus cells were then removed by gentle and repeated pipetting. Once they had been denuded, the mature good-quality oocytes were activated with two 60-μsec direct current (DC) pulses of 120 V/cm in 280 mM mannitol solution containing 0.01 mM CaCl_2_ and 0.05 mM MgCl_2_ using a BTX 2001 Electro-cell Manipulator (BTX, San Diego, CA, United States) for parthenogenetic activation (PA).

To produce SCNT embryos, the kidney cell of an aborted cloned male pig at 50 days of gestation was used as a primary culture of donor cells, cut into small pieces, and centrifuged several times. Culturing was performed in a 60-mm tissue culture plate in Dulbecco’s modified Eagle’s medium with 10% (v/v) fetal bovine serum until a monolayer of cells was completely formed. Donor cells at the G0/G1 stage of the cell cycle were synchronized for 48–72 h. For each replicate, the cells were passaged 3–8 times. Prior to nuclear transfer with the use of 0.4% (w/v) bovine serum albumin-TLH, donor cells were prepared by resuspension of trypsinized cultured cells. After 44 h of culturing in the maturation medium, COCs were denuded by gentle and repeated pipetting in 0.1% (wt/vol) hyaluronidase. Then, the denuded oocytes were incubated in 5 μg/mL Hoechst 33342 medium for 15 min and further treated with manipulation media overlaid with mineral oil. First, polar bodies were separated from metaphase II oocytes using a 17-μm beveled glass pipette. After enucleation, a single cell was inserted into the space between the zona pellucida and the membrane of the oocyte. Reconstructed SCNT oocytes were fused by electric cell fusion with two pulses of DC at 160 V/cm for 40 μs, followed by an alternating current of 2 V/cm for 2 s using a BTX 2001 Electro-cell Manipulator in 280 mM mannitol solution with a low Ca^+2^ concentration (0.001 mM). After 30 min of fusion, good-quality embryos were activated with two pulses of DC at 120 V/cm for 60 μs in 280 mM mannitol solution containing 0.01 mM CaCl_2_ and 0.05 mM MgCl_2_.

After electrical activation, both parthenogenic (PA) and cloned embryos were post-activated for 4 h with 10 μg/mL of cytochalasin B and 6-dimethylaminopurine. The embryos were then washed three times using *in vitro* culture medium (porcine zygote medium-5; IFP, Higashine, Yamagata, Japan) and then cultured in 25-μL droplets; pre-warmed mineral oil was used to cover the media, and embryos were cultured in 5% O_2_/5% CO_2_/90% N_2_ at 39°C in a humidified atmosphere. Embryo development and blastocyst development were assessed at days 2 and 6 for cleavage and blastocyst formation, respectively. Cell numbers were counted on day 6 blastocysts to determine the total cell number, the inner cell mass (ICM), and trophectoderm (TE) expression in accordance with differential staining protocol earlier reported in our previous work (Roy et al., 2020a).

### Expression of Genes Related to Nuclear Reprogramming

Embryos were harvested at different stages to compare the expression of genes related to nuclear reprogramming (i.e., *POU5F1*, *NDP52I1*, and *DPPA2*) by reverse transcriptase quantitative polymerase chain reaction (qRT-PCR). Total DNA was extracted using TRIzol reagent (Invitrogen Corporation) as described in our previous study (Kim et al., 2019). For expression analysis of the pluripotency genes *POU5F1*, *DPPA2*, and *NDP52IL*, total RNA was isolated from 6-day-old blastocysts of the control and treatment groups. Complementary DNA was synthesized from 300 ng of total RNA by qRT-PCR using 1 μL of DNA as a template and BIOFACT^TM^ 2 × Real-Time PCR Master Mix (BIOFACT Co., Ltd., Daejeon, South Korea) with the following reaction parameters: denaturation at 95°C for 15 min and 20 s, followed by 40 cycles of annealing and extension at 60°C for 40 s. The qRT-PCR primer sequences are presented in Table 1. The expression level of each target gene was quantified relative to that of the internal control gene (β-actin). The qRT-PCR specificity was determined via a melting curve analysis. Relative quantification was based on a comparison to the threshold cycle (Ct) at constant fluorescence intensity. Relative mRNA expression was quantified using the 2^–(ΔCt sample^^–^^ΔCt control)^ method (Livak and Schmittgen, 2001). To determine a normalized arbitrary value for each gene, every value was normalized to that of β-actin.

**TABLE 1:** Specific primers used for quantitative reverse transcription polymerase chain reaction for the gene expression analysis.

Gene	Sequence (5′–3′)	Product size (bp)	NCBI Accession No.
β-actin	F: CCC TGG AGA AGA GCT ACG AG R: TCC TTC CTG ATG TCC ACG TC	172	XM_003124280.5
*POU5F1*	F: AGT GAG AGG CAA CCT GGA GA R: TCG TTG CGA ATA GTC ACT GC	166	XM_021097869.1
*NDP52IL*	F: TGC TGA GTT ACA TGG GTC TGG R: ACC AAG GTC TGA TTT GCA GGT	182	XM_003131552.4
*DPPA2*	F: TGA GAG AGG GGA AAA GAC CAA R: TGG CAG AAA GGT CTC AAC AGA	151	XM_003358822.4

### Milrinone Treatment

Milrinone is a specific PDE inhibitor that has been shown to coordinate cytoplasmic and nuclear maturation in IVF (Nogueira et al., 2006). We presumed that it would have a beneficial effect on SCNT in coordinating cytoplasmic and nuclear maturation. Therefore, we identified and separated oocytes that seemed to require coordination of cytoplasmic and nuclear development. For this purpose, we considered various conventional oocyte selection criteria, with BCB staining as the categorization criteria, such that oocytes were classified into BCB+, BCB−, and control (without staining) (Figure 1). The BCB− oocytes were then randomly divided into four groups and treated with different concentrations of milrinone (0, 50, 75, or 100 μM) for 6 h; the control group (0 μM milrinone) was not exposed to milrinone but was placed directly in the IVM medium. The oocytes in each group were then placed in wells containing 500 μL of TCM-199 medium at 38.5°C in a humidified atmosphere of 5% CO_2_ and 95% air. The development rates of PA and SCNT embryos were evaluated.

### Experimental Designs

In experiment 1, the coordinated maturation of the cytoplasm with the nucleus was assessed as part of the developmental competence of oocytes. Developmental competence was related to the sizes of the ovarian follicles and the diameters of the oocytes, as evaluated by BCB staining (13 μM in mDPBS). The collected follicles were categorized into three groups according to their diameters: large (>5 mm), medium (3–5 mm), and small follicles (<3 mm). The percentage of select oocytes and the diameters of the oocytes were calculated according to their follicular size. Oocyte quality was also assessed by the higher GSH and lower ROS levels in incompetent oocytes. This experiment was repeated six times.

In experiment 2, the effects of coordinated cytoplasmic and nuclear maturation of oocytes were evaluated on the *in vitro* development of porcine PA and cloned embryos. The oocytes were categorized into those with well-coordinated cytoplasm and nuclear development or those needing coordination in IVM according to BCB staining (those not stained were the control group). Then, PA and SCNT were performed, and the effect of oocyte competence – essentially, the coordinated maturation of the cytoplasm with the nucleus – was analyzed by the competence of embryo development.

In experiment 3, we aimed to coordinate the cytoplasmic and nuclear maturation of oocytes in IVM, particularly in less competent oocytes that could be inefficient in coordinated cytoplasmic maturation. Therefore, we selected oocytes needing coordination (i.e., BCB− oocytes) and randomly divided them into four groups. Oocytes in the control group were placed directly into IVM medium, whereas those in the other three groups were treated individually with three different concentrations of milrinone (50, 75, and 100 μM) for 6 h. The *in vitro* development of PA and cloned embryos were then evaluated.

In experiment 4, the coordinated cytoplasmic and nuclear maturation of oocytes is essential not only for embryo cell cleavage and development but also for nuclear reprogramming. We aimed to evaluate the effect of cytoplasmic development, i.e., the attainment of *in vitro* coordinated maturation on the nuclear reprogramming efficiency in IVF and SCNT. The limitation in nuclear reprogramming is believed to be one of the major constraints in SCNT, and improving it would have a great impact on SCNT success. Therefore, we assessed the nuclear reprogramming efficiency of cytoplasmic and nuclear maturation coordination by milrinone though the evaluation of the expression levels of reprogramming-related genes *POU5F1*, *NDP52I1*, and *DPPA2* in SCNT oocytes.

### Statistical Analysis

Statistical Package for Social Sciences version 21.0 software (IBM, Armonk, NY, United States) was used to perform the data analysis. All values were presented as the mean ± standard error of the mean (SEM). Data related to oocyte maturation, blastocyst development rates in PA and cloned embryos, and cell number were analyzed using the generalized linear model procedure and one-way analysis of variance (ANOVA), and significance was determined using Duncan’s multiple range test. Significance in gene expression was also determined using ANOVA with Duncan’s multiple range test. GSH and ROS was analyzed by using *t*-test. Statistical significance was considered at *p* < 0.05.

## Results

### Selection of Developmentally Competent Oocytes

**FIGURE 2:**
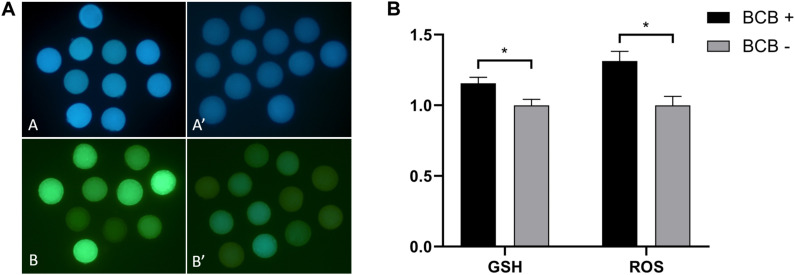
Epifluorescence photomicrographic images of *in vitro* mature porcine oocytes. **(A)** Glutathione (GSH; **A,A′**) and reactive oxygen species (ROS; **B,B′**) in oocytes in the brilliant cresyl blue-stained (BCB +) (**A,B**) and non-stained (BCB−) (**A′,B′**) oocyte groups. **(B)** Intracellular levels of GSH and ROS in *in vitro* mature porcine oocytes in the BCB + and BCB− oocyte groups. Values are means ± SEM (*n* = 8 replicates). Asterisks indicate *P* ≤ 0.05 when compared with each other.

The collected oocytes were assessed for coordinated cytoplasmic and nuclear maturation as an indicator of oocyte competence. The results showed that there was a significantly higher proportion of competent oocytes in large ovarian follicles (>5 mm) and medium ovarian follicles (3–5 mm), whereas the reverse was true for small ovarian follicles (<3 mm) (Figure 1). The mean diameter of denuded mature oocytes measured after IVM (including the zona pellucida) was significantly larger in competent oocytes than in less competent oocytes for large, medium, and small ovarian follicles, in alignment with follicular size. Similar to follicle size and oocyte diameter, GSH and ROS levels were significantly different between BCB + and BCB− oocytes (Figure 2).

### Development of PA and SCNT Embryos

BCB + oocytes showed significantly higher rates of maturation (87.5 ± 2.3% vs. 71.3 ± 3.0%) than BCB− oocytes (*p* < 0.05). Cleavage rates (88.6 ± 1.1% vs. 76.3 ± 1.6%) and blastocyst development (34.3 ± 0.9% vs. 25.3 ± 0.6%) were also significantly higher in BCB + oocytes than in control oocytes after PA, respectively (*p* < 0.05) (Table 2). Similarly, there were significantly higher cell numbers in the blastocysts (46.9 ± 1.4% vs. 38.9 ± 0.9%) of BCB + oocytes than in BCB− oocytes (*p* < 0.05).

**TABLE 2:** *In vitro* development of porcine parthenogenetic embryos derived from brilliant cresyl blue-stained (BCB +) and non-stained (BCB−) oocytes.

Group	No. of COCs	No. (%) of M-II oocytes	No. (%) of embryos		Total cell number in blastocysts
			Cleaved	Developed to BL	
Control	160	129 (80.6 ± 3.7)^*ab*^	107 (82.9 ± 2.1)^*ab*^	36 (27.8 ± 1.0)^*b*^	39.4 ± 0.7^*b*^
BCB +	160	140 (87.5 ± 2.3)^*a*^	124 (88.6 ± 1.1)^*a*^	48 (34.3 ± 0.9)^*a*^	46.9 ± 1.4^*a*^
BCB−	160	114 (71.3 ± 3.0)^*b*^	87 (76.3 ± 1.6)^*b*^	29 (25.3 ± 0.6)^*b*^	38.9 ± 0.9^*b*^

*Values in the same column with different superscript letters are significantly different (*p* < 0.05). Percentages are shown as means ± SEM (*n* = 4 replicates). BL, blastocyst; COC, cumulus–oocyte complex.*

In the case of cloned embryos, BCB + oocytes showed a significantly higher cleavage rate (77.9 ± 3.7% vs. 67.7 ± 2.3%) and blastocyst development rates (30.6 ± 1.6 vs. 20.1 ± 1.5) than those of BCB− oocytes (*p* < 0.05) (Table 3). The cell number in the blastocysts (46.7 ± 1.3% vs. 39.4 ± 2.0%) developed from BCB + oocytes was also significantly higher than that in BCB− oocytes (*p* < 0.05).

**TABLE 3:** *In vitro* development of porcine cloned embryos derived from brilliant cresyl blue-stained (BCB +) and non-stained (BCB−) oocytes.

No. (%) of embryos				

Group	Cultured	Cleaved	Developed to BL	Total cell number in blastocysts
Control	96	70 (72.9 ± 1.4)^*ab*^	25 (26.0 ± 0.8)^*ab*^	42.4 ± 2.0^*b*^
BCB +	108	84 (77.9 ± 3.7)^*a*^	33 (30.6 ± 1.6)^*a*^	46.7 ± 1.3^*a*^
BCB−	84	57 (67.7 ± 2.3)^*b*^	17 (20.1 ± 1.5)^*b*^	39.4 ± 2.0^*b*^

*Values in the same column with different superscript letters are significantly different (*p* < 0.05). Percentages are shown as means ± SEM (*n* = 4 replicates). BL, blastocyst; COC, cumulus–oocyte complex.*

### Milrinone Treatment of Oocytes and Embryo Production

*In vitro* maturation medium was supplemented with different concentrations of milrinone to enhance the coordination of nuclear meiosis with cytoplasm maturation. The results showed an increased rate of maturation of oocytes in all treated groups as compared to the untreated (73.0 ± 3.8%, 78.0 ± 2.6%, 76.0 ± 3.7% vs. 69.0 ± 2.5% for 50, 75, and 100 μM vs. 0 μM milrinone, respectively), although there was no statistically significant difference (Table 4). Furthermore, blastocyst development was significantly higher in the 75 μM treatment group than in the untreated control (38.5 ± 0.7% vs. 23.0 ± 1.6%) (*p* < 0.05). However, there was no significant difference in the total cell number in PA blastocysts among the different milrinone concentration treatments.

**TABLE 4:** *In vitro* development of porcine parthenogenetic embryos after treatment with four different milrinone concentrations in the brilliant cresyl blue non-stained (BCB−) oocytes.

Conc. of milrinone (μM)	No. of COCs	No. (%) of MII oocytes	No. (%) of embryos		Total cell number in blastocysts
			Cleaved	Developed to BL	
0	100	69 (69.0 ± 2.5)	52 (75.9 ± 5.0)	16 (23.0 ± 1.6)^*b*^	41.9 ± 1.9
50	100	73 (73.0 ± 3.8)	54 (74.2 ± 3.7)	21 (28.8 ± 2.4)^*b*^	43.4 ± 1.8
75	100	78 (78.0 ± 2.6)	66 (84.6 ± 3.0)	30 (38.5 ± 0.7)^*a*^	48.2 ± 1.6
100	100	76 (76.0 ± 3.7)	63 (82.7 ± 2.8)	23 (30.5 ± 2.3)^*ab*^	42.7 ± 1.6

*Values in the same column with different superscript letters are significantly different (*p* < 0.05). Percentages and the total cell number in BL are shown as means ± SEM (*n* = 4 replicates). BL, blastocyst; COC, cumulus–oocyte complex.*

In cloned embryo production, developmental competence was significantly improved in the 75 μM milrinone treatment group (30.1 ± 0.7% vs. 20.5 ± 2.2%) as compared to the control group (*p* < 0.05). However, there was no significant difference in blastocyst cell number among the four milrinone treatment groups (Table 5).

**TABLE 5:** *In vitro* development of porcine cloned embryos after treatment with four different milrinone concentrations in the brilliant cresyl blue non-stained (BCB−) oocytes.

Conc. of milrinone (μM)	No. (%) of embryos			Total cell number in blastocysts
	Cultured	Cleaved	Developed to BL	
0	58	47 (81.0 ± 1.7)	12 (20.5 ± 2.2)^*b*^	40.7 ± 1.8
50	60	50 (83.6 ± 2.4)	14 (23.6 ± 2.5)^*ab*^	43.4 ± 2.0
75	63	54 (85.9 ± 3.6)	19 (30.1 ± 0.7)^*a*^	47.1 ± 2.2
100	60	49 (81.7 ± 2.9)	16 (26.6 ± 2.4)^*ab*^	43.3 ± 2.1

*Values in the same column with different superscript letters are significantly different (*p* < 0.05). Percentages and the total cell number in BL are shown as means ± SEM (*n* = 6 replicates). BL, blastocyst; COC, cumulus–oocyte complex.*

### Nuclear Reprogramming in Milrinone-Treated Cloned Embryos

**FIGURE 3:**
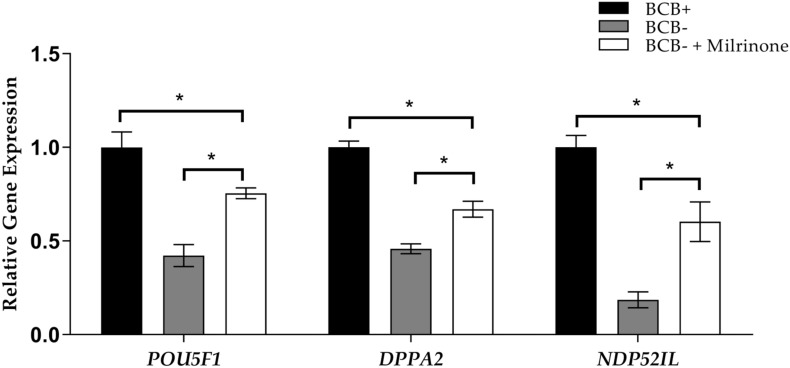
Relative expression levels of the *POU5F1*, *DPPA2*, and *NDP52IL* genes in cloned blastocysts derived from brilliant cresyl blue non-stained (BCB−) oocytes, BCB− oocytes supplemented with 75 μM milrinone, and BCB−stained (BCB +) oocytes. Asterisks indicate *P* ≤ 0.05 when compared with each other.

Gene expression analysis was performed to assess the role of coordinated cytoplasmic and nuclear maturation of oocytes in the efficiency of nuclear reprogramming in cloned porcine embryos. The expression of nuclear reprogramming-related genes *POU5F1*, *DPPA2*, and *NDP52IL* was analyzed in BCB + oocytes and BCB− milrinone-treated and non-treated oocytes. The results showed that gene expression was the highest in BCB + oocytes as compared to BCB− in both treated and non-treated groups. In contrast, milrinone treatment of the BCB− oocytes showed higher gene expression levels of *POU5F1*, *DPPA2*, and *NDP52IL* than BCB− non-treated oocytes (Figure 3).

## Discussion

In this study, we found that cytoplasmic maturation along with nuclear maturation is a critical factor in determining the developmental competence of oocytes. Most of the BCB + oocytes showed significantly higher developmental competence at the IVM level regarding embryo development and expression of genes related to nuclear reprogramming in cloned embryos. This indicates that cytoplasmic maturation is important for attaining developmental competence in cloned embryo production. Low competence oocytes (marked as BCB−) had low developmental competence, mainly owing to the absence of synchronized cytoplasmic and nuclear maturation, as cytoplasmic maturation required more time than nuclear maturation *in vitro*. In oocytes with less developmental competence, where cytoplasmic maturation is not aligned with nuclear maturation, there is a need to coordinate cytoplasmic maturation for efficient production of cloned embryos. Our experiment revealed that 75 μM milrinone during IVM improved the developmental competence of cloned embryos from BCB−- oocytes. BCB staining detects G6PDH activity in the oocyte cytoplasm and can be used to assess the competence of oocytes in terms of cytoplasmic maturation in alignment with nucleus maturation, as also previously described (Catala et al., 2011).

Milrinone supplementation in the IVM medium enhanced oocyte competence, with the development rates of both PA (38.5%) and cloned (30.1%) embryos being significantly higher than that of the control groups (23.0 and 20.5%, respectively). This is attributed to the delaying effect of milrinone as a specific PDE inhibitor. Similar results of milrinone as a PDE inhibitor were reported in mice (Giorgi et al., 2011), rats (Richard et al., 2001), and bovines (Naruse et al., 2012; Alam et al., 2018). Milrinone supplementation also tended to improve the maturation rate of the oocytes and the number of developed blastocysts. These results are consistent with those of previous studies in which sheep oocytes supplemented with milrinone showed improved developmental competence (Wang et al., 2016). The mechanism of milrinone, as a specific PDE3 inhibitor, is as follows: it downregulates PDE3, which affects the hydrolysis of cyclic nucleotides, cAMP and cGMP, in the cytoplasm of the oocytes (Gupta et al., 2020). Thus, the maturation promoting factors that trigger the meiotic exit of the oocyte are destabilized. In IVM, where the oocytes are aspirated from the follicle, they escape the oocyte maturation inhibitor in the follicular fluid. As a result, there is a spontaneous resumption of meiosis, where the nuclear and cytoplasmic maturation might not align. Therefore, the optimum amount of milrinone would be required to inhibit the spontaneous resumption of meiosis, allowing time for cytoplasmic maturation. The effectiveness of milrinone supplementation in improving the competence of oocytes is related to its antioxidant effects, which reduce oxidative damage, and its ability to maintain high levels of cAMP through PDE3 degradation (Wang et al., 2016). High levels of cAMP regulate downstream protein kinase (Wang et al., 2016), arresting the GV stage and ultimately blocking spontaneous meiotic resumption (Vanhoutte et al., 2007). Therefore, as seen in our study, milrinone caused a delay that was required for the coordination of cytoplasmic and nuclear maturation, positively influencing further development. This finding will have important applications for endangered wild species with few oocyte reserves or monotocous species such as humans, which have a low oocyte recovery rate.

Gene expression analysis for nuclear reprogramming showed significantly higher expression in BCB + oocytes than in BCB− oocytes. Milrinone treatment of BCB− oocytes moderately improved gene expression as compared to the non-treated BCB− oocytes. This increase in gene expression may have been due to modifications that occurred in the cytoplasm, such as increased levels of GSH and cAMP, resulting in a beneficial environment for the reprogramming of the donor nuclei. Thus, milrinone supplementation during IVM of oocytes can improve the efficiency of cloned pig embryo production. This will be extremely useful in addressing one of the core limitations of cloning and will improve nuclear reprogramming efficiency in pigs and other animal species.

In conclusion, we recommend that oocyte competence is the core issue in *in vitro* embryo production and cloning of porcine embryos, not only for coyote totipotency and cell multiplication but also for nuclear reprogramming. In IVM, coordination of cytoplasmic and nuclear maturation is required, particularly in less competent oocytes, to attain better efficiency in embryo production and cloning in pigs and other animal species. We recommend that 75 μM milrinone can be used for the coordination of cytoplasmic and nuclear maturation of oocytes to improve the efficiency of porcine SCNT.

## Data Availability Statement

The raw data supporting the conclusions of this article will be made available by the authors, without undue reservation.

## Author Contributions

PR and JC contributed to the study design. PR, AQ, BT, GK, SB, and JC contributed to data analysis. PR, XF, and JC performed the experiments. PR, AQ, BT, SB, and JC wrote the manuscript. JC acquired financial support for the project leading to this publication. All authors contributed to the article and approved the submitted version.

## Conflict of Interest

The authors declare that the research was conducted in the absence of any commercial or financial relationships that could be construed as a potential conflict of interest.
